# Neuroregeneration and dementia: new treatment options

**DOI:** 10.1186/2049-9256-1-12

**Published:** 2013-07-17

**Authors:** Smaranda Ioana Mitran, Bogdan Catalin, Veronica Sfredel, Tudor-Adrian Balseanu

**Affiliations:** Department of Functional Sciences, University of Medicine and Pharmacy, Craiova, Romania; Center of Clinical and Experimental Medicine, University of Medicine and Pharmacy, Craiova, Romania; Physiology Department, University of Medicine and Pharmacy, No 2 Petru Rares street, Craiova, Romania

**Keywords:** Neuroregeneration, Ageing, Dementia, Dementia therapy

## Abstract

In the last years, physiological aging became a general concept that includes all the changes that occur in organism with old age. It is obvious now, that in developing and developed countries, new health problems concerning older population appear. One of these major concerns is probably dementia. Sooner or later, all forms of dementia lead to learning deficit, memory loss, low attention span, impairment of speech and poor problem solving skills. Normal ageing is a physiological process that also involves a lot of neurological disorders with the same type of symptoms and effects that many researchers are trying to minimize in demented patients. In this review we try to highlight some of the newest aspects of therapeutic strategies that can improve natural neuroregeneration.

## Introduction

With longer life span, especially in developing and developed countries, new health problems concerning specific needs of older populations arise. One of the major health concerns in this aspect is dementia.

Dementia is a clinical term used to characterize a number of diseases with neuropsychiatric symptoms, that are not explained by delirium or major psychiatric disorder and interfere with one's ability to function with usual activities [1] but dementia as a biological aspect of the clinical term has many different causes.

All changes that occur in all organs and systems associated with old age is now generally accepted as physiological ageing. This concept is characterized by the declining ability to respond to different types of stress, homeostatic imbalance and diseases.

Brain ageing is associated with structures and functions decline both is neurons and glia. This is generally referred as neurodegeneration. It is a normal process that can lead to reductions in communication and in memory function [1, 2] but also to poor recovery after stroke [3].

Neurodegeneration is a progressive process that occurs because of a decline in the total number of neurons; generally, this process is due to apoptosis and is associated with a loss of neuronal structure and function [4]. Ageing is considered the most important risk factor for brain degeneration and age-related cognitive disorders [5].

Elucidating the cellular and molecular basis of neurodegeneration and neuroregeneration and how they are involved in the aging brain could reveal new therapeutic approaches to dementia in the elderly.

Overall, we aimed at summarizing up to-date findings described and evaluated by a number of different authors and by discussing contradictory results of different researcher groups we want to highlight the complexity of the presented problem.

## Review

### Neuroregeneration and aging

Physiological ageing is now generally accepted as a concept that includes all the changes that occur in all organs and systems associated with old age, characterized by the declining ability to respond to different types of stress, homeostatic imbalance and diseases.

The phenomena involved in brain ageing are associated with a decline of the neuronal and glial structures and functions, which can lead to reductions in communication and in memory function [1, 2] and also to poor recovery after stroke [3].

Neurodegeneration is a progressive process that occurs because of a decline in the total number of neurons; generally, this process is due to apoptosis and is associated with a loss of neuronal structure and function [4]. Ageing is considered the most important risk factor for brain degeneration and age-related cognitive disorders [5].

An increasing susceptibility to poor recovery from brain injury has been observed in the elderly. Regeneration of neuronal tissue makes no exception however the pathways for the cellular processes that characterize these phenomena have yet to be found.

From a morphological and functional perspective neuroregeneration implies a restorative process involving central nervous system tissue and neural network architecture. From a clinical point of view, brain-repairing pathways require the removal of the aetiological factors that caused the damage after the inflammatory response, to impede the degeneration of cells and to restore the neural cells or their network [6].

The concept of neuregeneration can be also defined as the superposition of three distinctive processes including neurogenesis, neuroplasticity and neurorestoration [7]. It is known that the mammalian brain is one of the organs with a very low level of cell replacement. Still, neurogenesis is now generally accepted and neural stem cells have been discovered in different specific brain regions, including hippocampus [8].

However, older organisms, including humans, have reported a decline in the rate of neurogenesis. Hippocampal neurogenesis seems to persist also in adult age. With ageing, the proliferation and differentiation of neural progenitors are significantly decreased with a higher number of newborn death neurons.

One possible explanation for this decline can be that the neural stem cells are entering in a quiescent state, supported by the hypothesis that aged neural stem cells are intrinsically different from the younger one. Another possibility can be that the expression of some transcription factors (Er81 and Dlx2) associated with neural development are reduced in the elderly [9].

Another effect can be the reduction in telomerase activity. A study on adult mice deficient in telomerase activity, reported poor neurogenesis in hippocampal areas, associated with a lower number of proliferation (Ki67+) or immature (Dcx+) cells [10].

Nevertheless, extrinsic factors from the local environment (neurogenic niche) can regulate neurogenesis. In old age, lower levels for different growth factors that can facilitate neurogenesis (fibroblast growth factor-2, insulin growth factor-1 or vascular endothelial growth factor) were registered in the hippocampal area [11]. A decline in angiogenesis in cerebrovascular area observed in aged mammals [12] can also modify the neurogenic niche by decreasing the source of growth factors.

### Ageing and dementia

As populations get older due to a longer life span, especially in developing and developed countries, new health problems concerning specific needs of older populations arise. One of the major health concerns in this aspect is dementia.

Dementia has many different causes. Dementia is seen as a clinical term used to characterize a number of diseases such as Alzheimer's disease dementia, Lewy bodies dementia, vascular dementia, behavior variant fronto-temporal dementia, primary progressive aphasia or any cognitive/behavioral changes with neuropsychiatric symptoms, that are not explained by delirium or major psychiatric disorder and interfere with one's ability to function with usual activities [1, 2].

At the moment it is estimated that 35.6 million people worldwide are diagnosed with dementia. It is predicted that in approximately 20 years this number will double and triple in the next 40 years [13].

**Figure 1:**
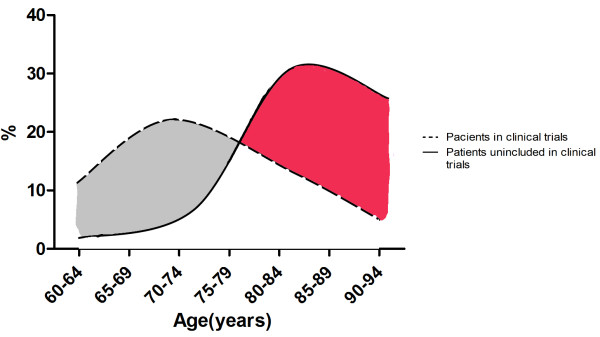
**Representation of age distribution for patients suffering from dementia.** Broken line shows patients that were included in clinical research of diagnostic methods and therapeutic trials. Solid line shows demented patients age distribution from the general population of the Netherlands [14].

Based on all the information the scientific community has gathered, the diagnostic criteria for dementia has became more accurate [1, 12]. Treatment and management of dementia are generated by research based on results from studies. Test subjects are patients with early stages of dementia Figure 1[14]. As the population gets older, new criteria for diagnosing old elderly persons (>75 years old) are needed because the border between non pathological aged brain and dementia brain is becoming more unclear with age [15].

As one gets older, multiple risk factors varying from childhood IQ [16], obesity in middle age [17], smoking, hypertension, high cholesterol and diabetes at midlife [18, 19] to stroke and atrophy in old age [15] contribute to dementia in the elderly. Scientific progress has found that a small number of highly conserved genes can have a positive effect on the length of a life span. These genes act through a number of metabolic processes like the “reactive oxygen species” (ROS) representing one of the most incriminated and poorly understood contributors to brain ageing [20]. Acquiring defense from dietary antioxidants can protect against ROS induced damage [16].

It is believed that the brain has a neuronal reserve to compensate for any loss at old age. However the cause of dementia is a more rapid neuronal death as found in alcohol abuse, unhealthy eating, smoking, stroke. Regardless of the risk factors, dementia is still a consequence of neuronal populations loss that cannot be compensated. It is unclear as to why there is such large individual variability in our population.

Studies done on old elderly persons have shown that as people get older, it is harder to distinguish between a normal aged brain and a dementia affected brain [15]. A constant increase in neuritic plaques and tangles in the hippocampus and neocortex of non-dementia diagnosis persons are parallel with constant decrees in the same features of a dementia brain. The only objective and quantifiable diagnostic criteria still remaining valid in both old elderly persons (> 75 years old) as of young elderly persons (< 75 years old) is cerebral atrophy [15]. Furthermore, neuropathological examination can sometimes detect individuals with microscopic features typical of late-onset Alzheimer’s but no clinical history of dementia was described before death. Other times, individuals diagnosed with late stage dementia during their life can show only mild pathological features of Alzheimer’s disease [16].

It is still not clear if cerebrovascular disease induces dementia or if both have the same risk factors and thus are indirectly linked. Despite all that, huge evidence associating stroke to dementia is simply undisputed, making it just more probable that stroke itself is a risk factor for dementia and not just a more acute consequence [21]. Studies on cerebrovascular system in the elderly populations have found that the blood brain barrier permeability increases with normal age, and can lead to the initiation or worsening of cerebral microvascular disease [22] or dementia.

An overlap can be found between vascular dementia and Alzheimer dementia. As previously noted [23], spontaneous cerebral emboli were detected in 43% of AD patients and 45% in VaD cases. These results were associated with a cognitive and functional decline within 2 years from the measurements. It was also found during this study that patients had an increase in psychiatric symptoms [24]. Most of these studies suggest that AD patients have a lower mean flow velocity than normal elderly patients [25, 26]. However some groups did not find any difference between AD patients and normal old persons [27, 28].

In the last 65 years, epidemiological studies have shown a double increase for the risk of being diagnosed with dementia [21]. The same studies determine that there is a risk of post-stroke dementia; this is dependent on the existence of pre-stroke cognitive impairment. Interestingly, stroke survivors who did not develope dementia symptoms at the age of 85 year have no increased risk of dementia compared to stroke free older persons. Neuropathological findings have shown that the burden and location of cerebral infarcts are associated with a cognitive decline [29]. However no evidence was found that stroke prevention treatments, especially antihypertensive tretments, have any effect on dementia incidence [30], although cohort studies found that statin treated patients seems to have a decrease risk of vascular dementia [31].

Understanding individual differences in age related cognitive decline and dementia is more difficult than we like to admit. The best available neuropathological evidence of the hypothetical difference between ‘normal’ ageing and dementia are scarce [16], suggesting there are no boundaries between them [15].

As molecular biology gets increasingly involved in the description of dementia, we hope that the border between different subtypes of dementia will slowly become clearer. By doing this we hope that we can find some means to postpone or even prevent dementia altogether.

### Treatment

“Early onset dementia”, a sub-type of dementia that occurs before the age of 65, or “late onset dementia”, more common among the geriatric population, both lead the patient to be disorientated in time, place and identity. Therefore, many studies seek other ways of renewing “the natural cargo” of the brains neurons, in order to remove various behavioral and psychological symptoms of dementia.

A promising approach regarding central nervous system degenerative diseases is a recombinant DNA vaccine composed of multiple specific inhibitory domains of NOIs (neurite outgrowth inhibitors). The mechanism of the treatment is an immunological one, inducing effective antibodies against the specific domains in the sera of mice treated with a DNA primed-vaccinia virus boost regimen. Three good responses were observed: little formation of soluble Aβ oligomer and amyloid plaques in the co-transgenic mice brain (incriminated in the physiopathology of Alzheimer’s disease), attenuated neuronal degeneration and protection against behavioral deficits [32]. The modulation of mRNA expression regarding NOIs was also obtained using generated channelrhodopsin-mutant protein expressing microglia, a sodium channel activated by light irradiation. By increasing the intracellular level of the ion, this technique can control microglial activation and thus cause repair/regeneration of neural and oligodendrocytic damage [33].

Stem cells might be a real alternative to brain regeneration in neurodegenerative disorders such as dementia, because they are capable of being integrated into a degenerative environment (the differentiation potential towards site appropriate phenotypes) and releasing neurotrophic cytokines. Quantification of the cytokines that may sustain endogenous neurogenesis and/or activate neuroprotective pathways (for nestin and connexin 43) revealed a neurogenic/angiogenic predisposition of naive human chorial villi and amniotic fluid derived cells. These cells also release significant amounts of brain-derived neurotrophic factors, as well as vascular endothelial growth factor [34].

Some ongoing clinical research trials, predict a chance for therapy using only neurotrophic factors: the neurotrophin nerve growth factor (NGF), glial cell-derived neurotrophic factor (GDNF) and brain-derived neurotrophic factor (BDNF). Already, some degree of success has been obtained with GDNF and NGF regarding the survival, existence and regeneration of specific neuronal populations in the adult brain suffering from Parkinson's or Alzheimer's disease [35, 36].

Some natural substances were also used to help neuronal self-healing. Among them, Cerebrolysin represents a therapeutic strategy for neurological disorders like dementia, stroke and traumatic brain injury. It is a peptide preparation mimicking the action of neurotrophic factors. Studies *in vitro* (biochemical and cell cultures) or *in vivo* (on animal models) show the multiple benefits of the natural drug (a pig brain extract). The drugs enhances neurogenesis in the dentate gyrus (the basis for neuronal replacement therapy in neurodegenerative diseases), but also in the subventricular zone (SVZ) sustaining the brain's self-repair. It also promotes neural progenitor cell migration, increases synaptic density rebuilding neuronal cytoarchitecture, induces restorative processes, decreases the infarct volume and edema formation and promotes functional recovery [37].

One study on neuronal precursor cells - Nestin+ and GFAP+ (glial fibrillary acidic protein) - isolated and cultured from adult rat SVZ, obtained good results using a pre-treatment of Quercetin in a dose–response manner. The natural flavonoid could be protective of neuronal precursors of adult brain by neutralizing the oxidative stress (reduces peroxynitrite formation, protein nitration and M2 isoform of pyruvate kinase depletion) [38]. Another flavonoid, isoquercitrin also promotes neuronal differentiation through multiple Rho GTPase mediated mechanisms. One possible mechanism is the inactivation of RhoA/Rho kinase: isoquercitrin reduces 47% of the RhoA activity and induces neurite growth (at concentrations ≥ 40 μM). Another mechanism is the affection of RhoA localization that underwent translocation to the cytoplasm [39].

Hormonal substances like the neurosteroid allopregnanolone (APα, 3α-hydroxy-5α-pregnan-20-one), a metabolite of progesterone that is normally generated in the nervous system, were also used for research with good results. In a triple transgenic mouse model of AD it promoted neurogenesis, improved the cognitive function and reversed the neurogenic deficits in the hippocampal dentate gyrus and the cerebral subventricular zone [40, 41].

Melatonin, the pineal product, has good antioxidant properties and has been shown to be useful in stopping neurodegenerative phenomena seen in experimental models of Alzheimer's disease, Parkinsonism and ischemic stroke. In clinical trials using treatments with melatonin has been effective in slowing the progression of Alzheimer's disease, but not of Parkinson's disease. Melatonin has multiple ways of preserving mitochondrial homeostasis. It reduces free radical generation by enhancing mitochondrial glutathione levels and it safeguards proton potential and ATP synthesis by stimulating complex I and IV activities [42].

## Conclusion

The available evidence indicates that one of the major health problems concerning the older population in developed countries will be dementia. It is a concern in the healthcare community that therapeutic strategies available do not address the cause of the pathology but are more focused on treating symptoms and providing support to patients.

Nevertheless, high rate of neurodegeneration is often found in elderly populations, further compromising the recovery of an injured brain.

Elucidating the cellular and molecular basis for involved in neurodegeneration and neuroregeneration in the aging brain could reveal new therapeutic approaches to dementia in the elderly.

Development of novel and effective therapies to sustain the brain's self-repair, using either molecular modulation of mRNA expression, stem cell therapy, neurotrophic factors or even drugs derived from natural sources will hopefully lead to an improved management of dementia in older population.

Multiple strategies should be targeted to prevent the development and the progression of the disease. Further investigation to update these strategies are required for an efficient therapeutic response in dementia.

## Abbreviations

ROS: Reactve oxygen species
AD: Alzheimer’s disease
NOIs: Neurite outgrowth inhibitors
NGF: Neurotrophin nerve growth factor
GDNF: Glial cell-derived neurotrophic factor
BDNF: Brain-derived neurotrophic factor brain-derived neurotrophic factor
SVZ: Subventricular zone
GFAP+: Fglial fibrillary acidic protein.
